# Identification of a putative molecular subtype of adult-type diffuse astrocytoma with recurrent MAPK pathway alterations

**DOI:** 10.1007/s00401-024-02766-2

**Published:** 2024-07-18

**Authors:** Philipp Sievers, Franck Bielle, Kirsten Göbel, Daniel Schrimpf, Lucia Nichelli, Bertrand Mathon, Romain Appay, Henning B. Boldt, Hildegard Dohmen, Carmen Selignow, Till Acker, Ales Vicha, Horacio Martinetto, Leonille Schweizer, Ulrich Schüller, Sebastian Brandner, Pieter Wesseling, Simone Schmid, David Capper, Zied Abdullaev, Kenneth Aldape, Andrey Korshunov, Sandro M. Krieg, Wolfgang Wick, Stefan M. Pfister, Andreas von Deimling, David E. Reuss, David T. W. Jones, Felix Sahm

**Affiliations:** 1 Department of Neuropathology, Institute of Pathology, University Hospital Heidelberg, Heidelberg, Germany; 2 Clinical Cooperation Unit Neuropathology, German Consortium for Translational Cancer Research (DKTK), German Cancer Research Center (DKFZ), Heidelberg, Germany; 3 Hopp Children’s Cancer Center Heidelberg (KiTZ), Heidelberg, Germany; 4 Sorbonne Université, Inserm, CNRS, UMR S 1127, Paris Brain Institute, ICM, 75013 Paris, France; 5 Department of Neuropathology, AP-HP, Pitié-Salpêtrière Hospital, 75013 Paris, France; 6 Department of Neuroradiology, AP-HP, Pitié-Salpêtrière Hospital, 75013 Paris, France; 7 Department of Neurosurgery, AP-HP, Pitié-Salpêtrière Hospital, 75013 Paris, France; 8 Department of Pathology and Neuropathology, APHM, CHU Timone, Marseille, France; 9 Institute of Neurophysiopathol, CNRS, INP, Aix-Marseille University, Marseille, France; 10 Department of Pathology, Odense University Hospital, Odense, Denmark; 11 Department of Clinical Research, University of Southern Denmark, Odense, Denmark; 12 Institute of Neuropathology, Justus-Liebig University Giessen, Giessen, Germany; 13 Prague Brain Tumor Research Group, Second Faculty of Medicine, Charles University and University Hospital Motol, Prague, Czech Republic; 14 Department of Pediatric Haematology and Oncology, Second Faculty of Medicine, Charles University and University Hospital Motol, Prague, Czech Republic; 15 Departamento de Neuropatología y Biología Molecular, Instituto de Investigaciones Neurológicas Dr Raúl Carrea (FLENI), Buenos Aires, Argentina; 16 Institute of Neurology (Edinger Institute), University Hospital Frankfurt, Goethe University, Frankfurt am Main, Germany; 17 German Cancer Consortium (DKTK), Partner Site Frankfurt/Mainz, German Cancer Research Center (DKFZ), Heidelberg, Germany; 18 Frankfurt Cancer Institute (FCI), Frankfurt am Main, Germany; 19 Department of Pediatric Hematology and Oncology, University Medical Center Hamburg-Eppendorf, Hamburg, Germany; 20 Research Institute Children’s Cancer Center Hamburg, Hamburg, Germany; 21 Institute of Neuropathology, University Medical Center Hamburg-Eppendorf, Hamburg, Germany; 22 Division of Neuropathology, National Hospital for Neurology and Neurosurgery, University College London Hospitals NHS Foundation Trust, Queen Square, London, UK; 23 Department of Neurodegenerative Disease, UCL Queen Square Institute of Neurology, Queen Square, London, UK; 24 Princess Máxima Center for Pediatric Oncology, Utrecht, The Netherlands; 25 Department of Pathology, Amsterdam University Medical Centers, Location VUmc and Brain Tumor Center Amsterdam, Amsterdam, The Netherlands; 26 Department of Neuropathology, Charité-Universitätsmedizin Berlin, Corporate Member of Freie Universität Berlin and Humboldt-Universität zu Berlin, Berlin, Germany; 27 Berlin Institute of Health, Charité-Universitätsmedizin Berlin, Berlin, Germany; 28 German Cancer Consortium (DKTK), Partner Site Berlin, German Cancer Research Center (DKFZ), Heidelberg, Germany; 29 Laboratory of Pathology, Center for Cancer Research, National Cancer Institute, National Institutes of Health, Bethesda, MD USA; 30 Department of Neurosurgery, Heidelberg University Hospital, Heidelberg, Germany; 31 Clinical Cooperation Unit Neurooncology, German Consortium for Translational Cancer Research (DKTK), German Cancer Research Center (DKFZ), Heidelberg, Germany; 32 Department of Neurology and Neurooncology Program, National Center for Tumor Diseases, Heidelberg University Hospital, Heidelberg, Germany; 33 Division of Pediatric Neurooncology, German Consortium for Translational Cancer Research (DKTK), German Cancer Research Center (DKFZ), Heidelberg, Germany; 34 Department of Pediatric Oncology, Hematology, Immunology and Pulmonology, University Hospital Heidelberg, Heidelberg, Germany; 35 National Center for Tumor Diseases (NCT), NCT Heidelberg, a partnership between DKFZ and Heidelberg University Hospital, Heidelberg, Germany; 36 Division of Pediatric Glioma Research, German Cancer Research Center (DKFZ), Heidelberg, Germany

Adult-type diffuse gliomas represent the most prevalent malignant primary tumors of the central nervous system (CNS), characterized by their highly invasive nature and therapy resistance [2, 6]. The 5th edition of the WHO classification of CNS tumors [5] classifies these gliomas primarily depending on the isocitrate dehydrogenase (*IDH1/2*) mutation status into three main types: IDH‐mutant astrocytoma; IDH‐mutant, 1p/19q co-deleted oligodendroglioma; and IDH‐wildtype glioblastoma. Recent progress in molecular profiling by DNA methylation analysis or next-generation sequencing has illuminated the vast heterogeneity of CNS tumors in particular in the pediatric context but also more broadly [5, 7]. These analyses have also identified attractive druggable targets for therapeutic intervention, including common alterations within the mitogen-activated protein kinase (MAPK) pathway in pediatric low-grade gliomas [4] and others.

Here, we explored a molecularly distinct subset of diffuse gliomas (*n* = 32), identified through unsupervised analysis of genome-wide DNA methylation data alongside copy number profiling and targeted next-generation sequencing. Application of unsupervised embedding analysis (t-SNE and UMAP) of DNA methylation profiles, alongside representative samples of established glioma types [1] unequivocally delineated these tumors as a distinct cluster (Fig. 1a, Supplementary Table 1 and 2, online resource). Analysis of copy number profiles derived from raw intensities of DNA methylation array probes revealed high-level amplification of *PDGFRA* in six of the 32 tumors. Amplification of *MYCN* (*n* = 2) and *CDK4* (*n* = 2) was observed in a smaller subset of cases. In one tumor, an *EGFR* amplification was found. Moreover, ten samples exhibited homozygous deletion of *CDKN2A/B*. Focal deletions in *NF1* (*n* = 4), *TP53* (*n* = 2) and *RB1* (*n* = 1) were infrequent. Importantly, none of the cases exhibited the combined chromosome 7 gain and chromosome 10 loss characteristic of IDH-wildtype glioblastoma or 1p/19q-codeletion as seen in oligodendrogliomas [5]. Evaluation of *MGMT* promoter methylation status, inferred from DNA methylation array data, revealed an unmethylated profile in almost all cases (31/32).

**Fig. 1:**
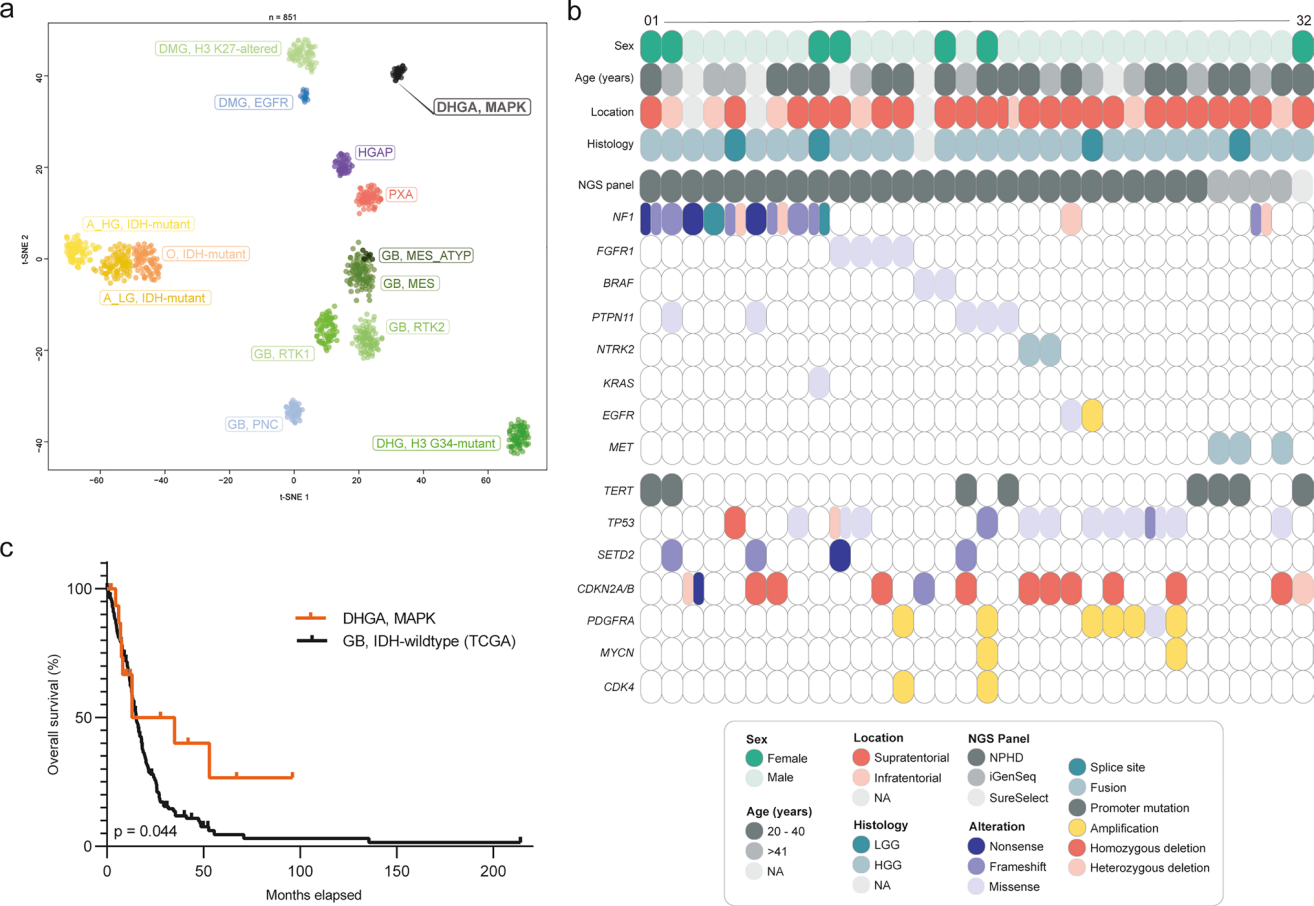
Unsupervised, nonlinear t-distributed stochastic neighbor embedding (t-SNE) projection of DNA methylation array profiles from 851 tumors (**a**). DNA methylation profiling reveals a molecular distinct group of diffuse high-grade astrocytomas (DHGA, MAPK; *n* = 32). For DNA methylation class abbreviations, see Supplementary Table 2. Clinicopathological characteristics and recurrent genetic alterations of the 32 diffuse gliomas (**b**). Kaplan–Meier curve for overall survival of 17 patients from the investigated series compared to a TCGA cohort of 230 IDH-wildtype glioblastoma patients (**c**)

Panel sequencing conducted on all 32 cases unveiled a significant prevalence of MAPK pathway alterations (Fig. 1b), including ten tumors with *NF1* mutations, four with *FGFR1* hotspot mutations (p.N546K or p.K656E), two with *BRAF* V600E mutation, and five with *PTPN11* mutation. Mutations in *EGFR* (p.A289T) and *KRAS* (p.V14I) were each seen in one case. Additionally, *TERT* promoter mutations were detected in eight tumors, alongside frequent alterations in *TP53* (*n* = 12), *BCOR* (*n* = 5), *SETD2* (*n* = 4), and ATRX (*n* = 3). Two tumors harbored an *NTRK2* fusion (*WNK2::NTRK2*, *NACC2::NTRK2*) and three a *PTPRZ1::MET* fusion. A comprehensive overview of the relevant variants detected is provided in Supplementary Table 3 (online resource). These findings highlight an enrichment of MAPK pathway alterations (78%) within this newly identified tumor subgroup, exhibiting a spectrum of alterations distinct from those commonly observed in other adult-type high-grade gliomas [5]. Given the availability of a variety of potent inhibitors targeting many of these alterations, this presents an enticing avenue for targeted therapeutic interventions [4].

Tumors within this series were predominantly located in the supratentorial compartment (*n* = 23), with only single cases occurring infratentorially or in the spinal region (Fig. 1b). Patient age at diagnosis ranged from 17 to 78 years, with a median age of 32 years. Clinical outcome data (overall survival) were available for 17 patients, showing a median survival time of 24 months. Among these patients, nine (53%) have deceased. Compared to a TCGA cohort of 230 IDH-wildtype glioblastoma patients, this group exhibited a marginally more favorable survival rate (*p* = 0.044; Fig. 1c). A small subset of patients (*n* = 4) initially exhibited lower-grade gliomas several years ago. Although a clear progression toward high-grade glioma was not universally documented for these tumors, the data suggest a possible progressive disease, as observed in other diffuse gliomas. Additionally, for one patient, a diagnosis of neurofibromatosis type 1 was known. Lack of material did not allow for a full assessment of potential links to tumor predisposition syndromes although this should be explored further in future given the high frequency of alterations affecting *NF1* and *PTPN11*.

The majority of tumors within this cohort were initially diagnosed histologically as high-grade glioma (27/32), predominantly glioblastoma, with a minority exhibiting lower-grade histological features. Histopathological review of 15 tumors revealed a relatively wide morphological spectrum of high-grade gliomas. All reviewed tumors shared a moderate to high increase in cellular density within a mostly fine fibrillary matrix. Tumors presented as polymorphous populations of predominantly astrocytic tumor cells with a variable degree of nuclear pleomorphism, often showing areas with giant cells and multinucleated cells with multiple tightly packed nuclei (Fig. 2a–d). Four of the cases exhibited focal oligodendroglial morphology with perinuclear halos due to cytoplasmic clearing (Fig. 2e). In one case, perivascular pseudorosettes and pleomorphic spindled cells were observed, at least focally. Ribbon-like structures were observed in one case. Calcifications were present in five tumors. Microvascular proliferation and necrosis were common across the majority of tumors, which mostly displayed a diffuse infiltrative pattern. Immunohistochemical analysis demonstrated immunoreactivity for OLIG2, with most cases showing diffuse positivity for GFAP (Fig. 2f, g). All analyzed tumors showed strong expression of vimentin, tested negative for synaptophysin and had restricted expression of CD34 to the vessels. Nuclear ATRX expression was preserved except in one case that harbored a nonsense variant (p.L389*). H3K27me3 was retained. Tumor cells enmeshed among axons stained for neurofilament protein, illustrating the infiltrative nature of the tumors (Fig. 2h). The Ki-67 labeling index varied between cases, with those displaying lower-grade features showing only 3–5% and higher-grade tumors showing up to 40% positivity on average (Supplementary Table 4, online resource).

**Fig. 2:**
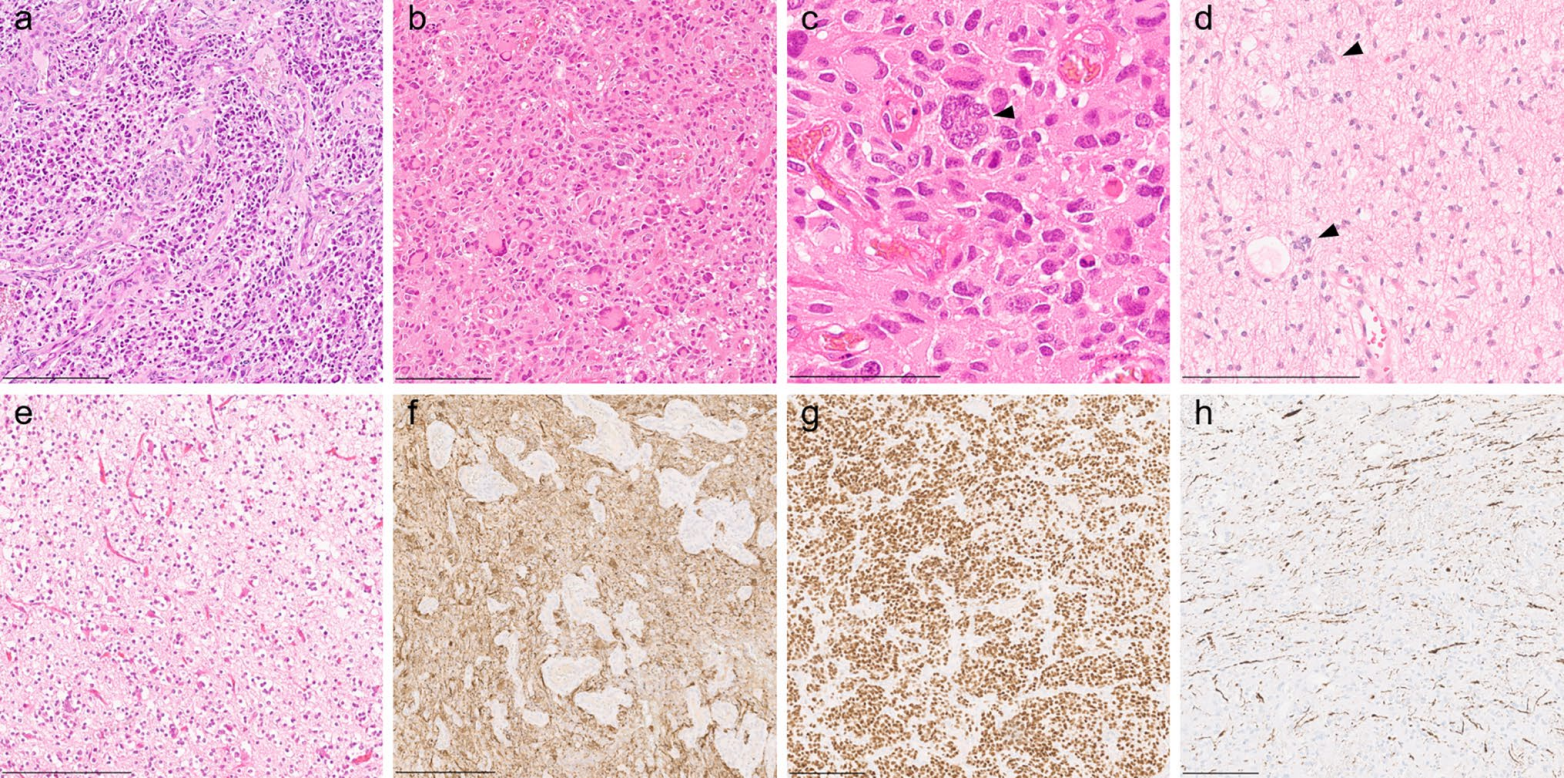
Hematoxylin and eosin (H&E) stainings showing pleomorphic astrocytic neoplasm with microvascular proliferation (**a**), giant cells (**b**) and multinucleated cells with multiple tightly packed nuclei (**c**). Lower-grade tumor with a fine fibrillary matrix and cells exhibiting multiple tightly packed nuclei (**d**). An example of a tumor showing perinuclear halos due to cytoplasmic clearing (e). Immunohistochemical staining for GFAP (**f**) and Olig2 (**g**) show strong positivity. Tumor cells infiltrate between axons stained for neurofilament protein (**h**). Scale bars: 200 μm

In summary, our investigation has revealed a distinct subtype of adult-type diffuse astrocytoma through DNA methylation profiling, lacking both *IDH1/2* mutation or chromosome + 7/-10 signature, but characterized by recurrent alterations within the MAPK pathway and with *TERT* promoter mutation in 25% of these neoplasms. Given the presence of targetable gene fusions within these tumors, RNA sequencing could be of value. While DNA methylation profiling has emerged as a pivotal tool in identifying novel CNS tumors, it is evident that sole reliance on epigenetic signatures may not be adequate for establishing new tumor types. The histopathological and molecular overlap with IDH-wildtype glioblastoma indicates that recognizing these tumors as an entirely new tumor type is not justified at this stage. This also implies that currently DNA methylation profiling remains the primary method for identifying these tumors, similar to other epigenetically defined tumor types [3, 8]. However, the notable prevalence of targetable MAPK alterations and the slightly more favorable survival rate compared to typical IDH-wildtype glioblastomas suggest that recognizing these tumors, at least provisionally, as a molecular subtype of IDH-wildtype glioblastomas may be valuable. We suggest the term ‘diffuse high-grade astrocytoma, MAPK pathway-altered’ to describe this molecular subtype of tumors. Further accumulation of cases and data is necessary to substantiate this distinction and understand the full clinical implications.

### Supplementary Information

Below is the link to the electronic supplementary material.

Supplementary file1 (XLSX 25 kb)

Supplementary file2 (DOCX 55 kb)

## Data Availability

The data that support the findings of this study are available from the corresponding author upon reasonable request.
