# Supplementary material for: Identification of a putative molecular subtype of adult-type diffuse astrocytoma with recurrent MAPK pathway alterations
Source: Acta Neuropathol. 2024 Jul 18;148(1):7. doi: 10.1007/s00401-024-02766-2 (PMC11258072; doi:10.1007/s00401-024-02766-2)
Supplement: Supplementary file 2 — Supplementary file2 (DOCX 55 kb) [file 401_2024_2766_MOESM2_ESM.docx]

**Supplementary materials and methods**

**Sample collection**

Patient tumor samples and retrospective clinical information were provided by multiple national and international collaborating centers and collected at the Department of Neuropathology of the University Hospital Heidelberg (UKHD, Germany) and German Cancer Research Center Heidelberg (DKFZ, Germany). Sample selection was based on unsupervised visualization (t-distributed stochastic neighbor embedding (t-SNE) and uniform manifold approximation and projection (UMAP)) of genome-wide DNA methylation array data that revealed a molecularly distinct group of tumors forming a cluster separate from all established tumor types. Furthermore, DNA methylation array data of numerous well-characterized reference samples representing CNS tumors were used for comparative analyses. Detailed descriptions of the reference DNA methylation classes are outlined under (<https://www.molecularneuropathology.org>). Sample collection and processing and data collection were performed in accordance with local ethics regulations and approval (ethical vote S-318/2022).

**DNA and RNA extraction**

Tumor DNA and RNA of samples processed in Heidelberg were extracted from areas with highest tumor cell content using the automated Maxwell system (Promega, Madison, WI, USA). Genomic DNA was extracted from fresh frozen or formalin-fixed and paraffin-embedded (FFPE) tissue samples with the Maxwell 16 Tissue DNA Purification Kit or the Maxwell 16 FFPE Plus LEV DNA Purification Kit (Promega), according to the manufacturer’s instructions. RNA was extracted from FFPE tissue samples by following the Maxwell 16 LEV RNA FFPE Kit protocol (Promega). Nucleic acid concentrations were determined using the Invitrogen Qubit dsDNA BR Assay Kit (Thermo Fisher Scientific, Waltham, MA, USA) on a FLUOstar Omega Microplate Reader (BMG Labtech, Ortenberg, Germany). For a subset of external cases, nucleic acid extraction was performed according to standard local procedures with corresponding QC measures.

**DNA methylation array processing and copy‑number profiling**

Genome-wide DNA methylation profiling of all samples was performed using the Infinium MethylationEPIC (EPIC) BeadChip (Illumina, San Diego, CA, USA) or Infinium HumanMethylation450 (450k) BeadChip array (Illumina) according to the manufacturer’s instructions and as previously described [3]. Raw data were generated at the Department of Neuropathology UKHD, the Genomics and Proteomics Core Facility of the DKFZ or at respective international collaborator institutes, using both fresh-frozen and FFPE tissue samples. All computational analyses were performed in R version 4.6.1 (R Development Core Team, 2020; <https://www.R-project.org>). *O6-methylguanine-DNA methyltransferase* (MGMT)-promoter methylation status was evaluated using the method described by Bady et al. [2]. Copy-number variation analysis from 450k and EPIC methylation array data was performed using the conumee Bioconductor package version1.12.0. Focal copy number alterations were called based on manual review of the log2 ratio plots for each sample. Raw signal intensities were obtained from IDAT-files using the minfi Bioconductor package version 1.21.4 [1]. Illumina EPIC and 450k samples were merged to a combined data set by selecting the intersection of probes present on both arrays (combineArrays function, minfi). Each sample was individually normalized by performing a background correction (shifting of the 5% percentile of negative control probe intensities to 0) and a dye-bias correction (scaling of the mean of normalization control probe intensities to 10,000) for both color channels. Subsequently, a correction for the array type (450k/EPIC) was performed by fitting univariable, linear models to the log2-transformed intensity values (removeBatchEffect function, limma package version 3.30.11). The methylated and unmethylated signals were corrected individually. Beta-values were calculated from the retransformed intensities using an offset of 100 (as recommended by Illumina). All samples were checked for duplicates by pairwise correlation of the genotyping probes on the 450k/EPIC array. To perform unsupervised non-linear dimension reduction, the remaining probes after standard filtering [3] were used to calculate the 1-variance weighted Pearson correlation between samples. The resulting distance matrix was used as input for t-SNE analysis (Rtsne package version 0.13). The following non-default parameters were applied: is_distance = T, theta = 0, pca = F, max_iter = 10,000 perplexity = 30.

**Targeted next-generation DNA sequencing**

DNA sequencing was performed in all tumor samples (n = 32). The majority of cases (27/32, 84%) were sequenced on a NovaSeq 6000 instrument (Illumina) at the Department of Neuropathology UKHD as previously described [6] using a customized enrichment/hybrid-capture-based next-generation sequencing (NGS) gene panel that comprised the entire coding (all exons+ /– 25 bp) and selected intronic and promoter regions of 170 genes of particular relevance in CNS tumors (NPHD2022A). Paired-end sequencing was applied to increase the detection sensitivity of duplicates and possible gene fusions. Sequence reads were mapped to the reference human genome build GRCh37 (hg19) using the Burrows–Wheeler aligner (BWA). For four cases (#28-31), mutational analyses were performed using custom-targeted NGS DNA sequencing panel at IGenseq of the Paris Brain Institute, as previously described [8]. One case (#32) was analyzed using a SureSelect CD Curie CGP panel at Institut Curie in Paris, France.

**RNA sequencing and analysis**

RNA sequencing for the purpose of gene fusion detection was performed in 8/32 of the cases. For six cases RNA sequencing (NPHDRNA) was performed on a NovaSeq 6000 instrument (Illumina) as previously described [7]. In brief, RNA sequencing libraries were prepared using the TruSeq RNA Library Prep for Enrichment kit (Illumina) and paired-end reads were sequenced. After adapter trimming, reads were aligned to the human genome (GRCh37) with the STAR aligner [4]. Fastq files from transcriptome sequencing were used for de novo annotation of fusion transcripts using the Arriba (v1.2.0) algorithm [9] with standard parameters, which removes recurrent alignment artifacts, transcript variants also observed in normal tissue, reads with low sequence complexity, and events with short anchors or breakpoints in close proximity or a low number of supporting reads relative to the overall number of predicted events in a gene. In addition, five cases (#28-32) were analyzed at the Institut Curie Paris using different archer fusion panels (Supplementary Table 1, online resource).

**Histology and immunohistochemistry**

A histopathological review based on hematoxylin-eosin (H&E) slides was retrospectively performed for 15 samples to investigate the morphological features of tumors within the novel group. Due to the aspect of a multicenter cohort, availability of tissue was restricted for some of the cases. Additional immunohistochemical stainings were performed for a subset of tumors (Supplementary Table 4, online resource). H&E and immunohistochemical staining was either performed at the UKHD Department of Neuropathology or received from the respective collaborator institutes. Immunohistochemical staining was performed on a Ventana BenchMark ULTRA Immunostainer using the ultraView Universal DAB Detection Kit (Ventana Medical Systems, Tucson, AZ, USA). Antibodies were directed against: glial fibrillary acid protein (GFAP; Z0334, rabbit polyclonal, 1:1000 dilution, Dako Agilent, Santa Clara, CA, USA), oligodendrocyte lineage transcription factor 2 (OLIG2; clone EPR2673, rabbit monoclonal, 1:50 dilution, Abcam, Cambridge, UK), ATRX (clone BSB-108, mouse monoclonal, 1:2000 dilution, Bio SB, Santa Barbara, CA, USA), anti-H3K27me3 (rabbit polyclonal, 1:100 dilution, Millipore, Burlington, MA, USA), Synaptophysin (clone MRQ-40, rabbit monoclonal, 1:160 dilution, Cell Marque Corp., Rocklin, CA, USA), CD34 (clone QBEnd/10, mouse monoclonal, Ventana Medical Systems), Neurofilament (clone 2F11, mouse monoclonal, 1:200 dilution, Dako Agilent), Vimentin (clone V9, mouse monoclonal, 1:900 dilution, Dako Agilent), and Ki-67 (clone MIB-1, mouse monoclonal, 1:100 dilution, Dako Agilent).

**Survival analysis**

Survival analysis was performed using GraphPad Prism 9 (GraphPad Software, La Jolla, CA, USA). Retrospective survival data were available for 17 patients in the series and were compared to a TCGA cohort of 230 IDH-wildtype glioblastoma patients (<https://www.cancer.gov/tcga>) [5]. Overall survival was defined as the period from the date of diagnostic surgery to the date of death. Kaplan-Meier curves were compared using the log-rank test, and a *p*-value of < 0.05 was considered statistically significant.

**References to supplementary methods**

1. Aryee MJ, Jaffe AE, Corrada-Bravo H, Ladd-Acosta C, Feinberg AP, Hansen KD et al. (2014) Minfi: a flexible and comprehensive Bioconductor package for the analysis of Infinium DNA methylation microarrays. Bioinformatics 30:1363-1369. doi:10.1093/bioinformatics/btu049

2. Bady P, Sciuscio D, Diserens AC, Bloch J, van den Bent MJ, Marosi C et al. (2012) MGMT methylation analysis of glioblastoma on the Infinium methylation BeadChip identifies two distinct CpG regions associated with gene silencing and outcome, yielding a prediction model for comparisons across datasets, tumor grades, and CIMP-status. Acta Neuropathol 124:547-560. doi:10.1007/s00401-012-1016-2

3. Capper D, Jones DTW, Sill M, Hovestadt V, Schrimpf D, Sturm D et al. (2018) DNA methylation-based classification of central nervous system tumours. Nature 555:469-474. doi:10.1038/nature26000

4. Dobin A, Davis CA, Schlesinger F, Drenkow J, Zaleski C, Jha S et al. (2013) STAR: ultrafast universal RNA-seq aligner. Bioinformatics 29:15-21. doi:10.1093/bioinformatics/bts635

5. Hai L, Hoffmann DC, Wagener RJ, Azorin DD, Hausmann D, Xie R et al. (2024) A clinically applicable connectivity signature for glioblastoma includes the tumor network driver CHI3L1. Nat Commun 15:968. doi:10.1038/s41467-024-45067-8

6. Sahm F, Schrimpf D, Jones DT, Meyer J, Kratz A, Reuss D et al. (2016) Next-generation sequencing in routine brain tumor diagnostics enables an integrated diagnosis and identifies actionable targets. Acta Neuropathol 131:903-910. doi:10.1007/s00401-015-1519-8

7. Stichel D, Schrimpf D, Casalini B, Meyer J, Wefers AK, Sievers P et al. (2019) Routine RNA sequencing of formalin-fixed paraffin-embedded specimens in neuropathology diagnostics identifies diagnostically and therapeutically relevant gene fusions. Acta Neuropathol 138:827-835. doi:10.1007/s00401-019-02039-3

8. Tran S, Thomas A, Aliouat I, Karachi C, Lozano F, Mokhtari K et al. (2023) A threshold for mitotic activity and post-surgical residual volume defines distinct prognostic groups for astrocytoma IDH-mutant. Neuropathol Appl Neurobiol 49:e12928. doi:10.1111/nan.12928

9. Uhrig S, Ellermann J, Walther T, Burkhardt P, Frohlich M, Hutter B et al. (2021) Accurate and efficient detection of gene fusions from RNA sequencing data. Genome Res 31:448-460. doi:10.1101/gr.257246.119
